# Refining motifs by improving information content scores using neighborhood profile search

**DOI:** 10.1186/1748-7188-1-23

**Published:** 2006-11-27

**Authors:** Chandan K Reddy, Yao-Chung Weng, Hsiao-Dong Chiang

**Affiliations:** 1 School of Electrical and Computer Engineering, Cornell University, Ithaca, NY, 14853, USA

## Abstract

The main goal of the motif finding problem is to detect novel, over-represented unknown signals in a set of sequences (e.g. transcription factor binding sites in a genome). The most widely used algorithms for finding motifs obtain a generative probabilistic representation of these over-represented signals and try to discover profiles that maximize the information content score. Although these profiles form a very powerful representation of the signals, the major difficulty arises from the fact that the best motif corresponds to the global maximum of a non-convex continuous function. Popular algorithms like Expectation Maximization (EM) and Gibbs sampling tend to be very sensitive to the initial guesses and are known to converge to the nearest local maximum very quickly. In order to improve the quality of the results, EM is used with multiple random starts or any other powerful stochastic global methods that might yield promising initial guesses (like projection algorithms). Global methods do not necessarily give initial guesses in the convergence region of the best local maximum but rather suggest that a promising solution is in the neighborhood region. In this paper, we introduce a novel optimization framework that searches the neighborhood regions of the initial alignment in a systematic manner to explore the multiple local optimal solutions. This effective search is achieved by transforming the original optimization problem into its corresponding dynamical system and estimating the practical stability boundary of the local maximum. Our results show that the popularly used EM algorithm often converges to sub-optimal solutions which can be significantly improved by the proposed neighborhood profile search. Based on experiments using both synthetic and real datasets, our method demonstrates significant improvements in the information content scores of the probabilistic models. The proposed method also gives the flexibility in using different local solvers and global methods depending on their suitability for some specific datasets.

## 1 Introduction

Recent developments in DNA sequencing have allowed biologists to obtain complete genomes for several species. However, knowledge of the sequence does not imply the understanding of how genes interact and regulate one another within the genome. Many transcription factor binding sites are highly conserved throughout the sequences and the discovery of the location of such binding sites plays an important role in understanding gene interaction and gene regulation.

**Figure 1:**
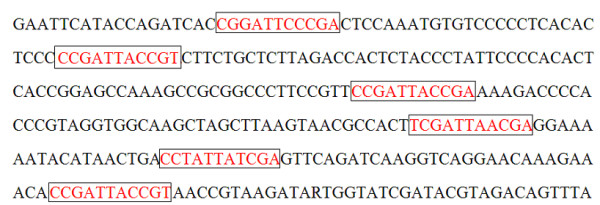
Synthetic DNA sequences containing some instance of the pattern 'CCGATTACCGA' with a maximum number of 2 mutations. The motifs in each sequence are highlighted in the box. We have a (11,2) motif where 11 is the length of the motif and 2 is the number of mutations allowed.

We consider a precise version of the motif discovery problem in computational biology as discussed in [1,2]. The planted (l, d) motif problem [2] considered in this paper is described as follows: Suppose there is a fixed but unknown nucleotide sequence *M*(the *motif*) of length *l*. The problem is to determine *M*, given *t *sequences with *t*_*i *_being the length of the *i*^*th *^sequence and each containing a planted variant of *M*. More precisely, each such planted variant is a substring that is *M *with exactly *d *point substitutions (see Fig. 1). More details about the complexity of the motif finding problem is given in [3]. A detailed assessment of different motif finding algorithms was published recently in [4].

Although there are several variations of the motif finding algorithms, the problem discussed in this paper is defined as follows: without any previous knowledge of the consensus pattern, discover all the occurences of the motifs and then recover a pattern for which all of these instances are within a given number of mutations (or substitutions). Despite the significant amount of literature available on the motif finding problem, many do not exploit the probabilistic models used for motif refinement [5,6].

We provide a novel optimization framework for refining motifs using systematic subspace exploration and neighborhood search techniques. This paper is organized as follows: Section 2 gives some relevant background about the existing approaches used for finding motifs. Section 3 describes the problem formulation in detail. Section 4 discusses our new framework and Section 5 details our implementation. Section 6 gives the experimental results from running our algorithm on synthetic and real datasets. Finally, Section 7 concludes our discussion with future research directions.

## 2 Relevant Background

Existing approaches used to solve the motif finding problem can be classified into two main categories [7]. The first group of algorithms utilizes a generative probabilistic representation of the nucleotide positions to discover a consensus DNA pattern that maximizes the information content score. In this approach, the original problem of finding the best consensus pattern is formulated as finding the global maximum of a continuous non-convex function. The main advantage of this approach is that the generated profiles are highly representative of the signals being determined [8]. The disadvantage, however, is that the determination of the "best" motif cannot be guaranteed and is often a very difficult problem since finding global maximum of any continuous non-convex function is a challenging problem. Current algorithms converge to the nearest local optimum instead of the global solution. Gibbs sampling [5], MEME [6], greedy CONSENSUS algorithm [9] and HMM based methods [10] belong to this category.

The second group uses patterns with 'mismatch representation' which define a signal to be a consensus pattern and allow up to a certain number of mismatches to occur in each instance of the pattern. The goal of these algorithms is to recover the consensus pattern with the most significant number of instances, given a certain background model. These methods view the representation of the signals as discrete and the main advantage of these algorithms is that they can guarantee that the highest scoring pattern will be the global optimum for any scoring function. The disadvantage, however, is that consensus patterns are not as expressive of the DNA signal as profile representations. Recent approaches within this framework include Projection methods [1,11], string based methods [2], Pattern-Branching [12], MULTIPROFILER [13] and other branch and bound approaches [7,14].

A hybrid approach could potentially combine the expressiveness of the profile representation with convergence guarantees of the consensus pattern. An example of a hybrid approach is the Random Projection [1] algorithm followed by EM algorithm [6]. It uses a global solver to obtain promising alignments in the discrete pattern space followed by further local solver refinements in continuous space [15,16]. Currently, only few algorithms take advantage of a combined discrete and continuous space search [1,7,11]. In this paper, the profile representation of the motif is emphasized and a new hybrid algorithm is developed to escape out of the local maxima of the likelihood surface.

Some motivations to develop the new hybrid algorithm proposed in this paper are :

• A motif refinement stage is vital and popularly used by many pattern based algorithms (like PROJECTION, MITRA etc) which try to find optimal motifs.

• The traditional EM algorithm used in the context of the motif finding converges very quickly to the nearest local optimal solution (within 5–8 iterations).

• There are many other promising local optimal solutions in the close vicinity of the profiles obtained from the global methods.

In spite of the importance placed on obtaining a global optimal solution in the context of motif finding, little work has been done in the direction of finding such solutions [17]. There are several proposed methods to escape out of the local optimal solution to find better solutions in machine learning [18] and optimization [19] related problems. Most of them are stochastic in nature and usually rely on perturbing either the data or the hypothesis. These stochastic perturbation algorithms are inefficient because they will sometimes miss a neighborhood solution or obtain an already existing solution. To avoid these problems, we introduce a novel optimization framework that has a better chance of avoiding sub-optimal solutions. It systematically escapes out of the convergence region of a local maximum to explore the existence of other nearby local maxima. Our method is primarily based on some fundamental principles of finding exit points on the stability boundary of a nonlinear continuous function. The underlying theoretical details of our method are described in [20,21].

## 3 Preliminaries

We will first describe our problem formulation and the details of the EM algorithm in the context of motif finding problem. We will then describe some details of the dynamical system of the log-likelihood function which enables us to search for the nearby local optimal solutions.

### 3.1 Problem Formulation

Some promising initial alignments are obtained by applying projection methods or random starts on the entire dataset. Typically, random starts are used because they are cost efficient. The most promising sets of alignments are considered for further processing. These initial alignments are then converted into profile representation.

Let *t *be the total number of sequences and *S *= {*S*_1_, *S*_2_...*S*_*t*_} be the set of *t *sequences. Let *P *be a single alignment containing the set of segments {*P*_1_, *P*_2_, ..., *P*_*t*_}. *l *is the length of the consensus pattern. For further discussion, we use the following variables

*i *= 1 ... *t *    - - for *t *sequences

*k *= 1 ... *l *    - - for positions within an *l*-mer

*j *∈ {*A*, *T*, *G*, *C*}     - - for each nucleotide

The count matrix can be constructed from the given alignments as shown in Table 1. We define *C*_0, *j *_to be the overall background count of each nucleotide in all of the sequences. Similarly, *C*_*k*, *j *_is the count of each nucleotide in the *k*^*th *^position (of the *l - mer*) in all the segments in *P*.

**Table 1:** Position Count Matrix.

*j*	*k *= 0	*k *= 1	*k *= 2	*k *= 3	*k *= 4	...	*k = l*
*A*	*C*_0,1_	*C*_1,1_	*C*_2,1_	*C*_3,1_	*C*_4,1_	...	*C*_*l*,1_
*T*	*C*_0,2_	*C*_1,2_	*C*_2,2_	*C*_3,2_	*C*_4,2_	...	*C*_*l*,2_
*G*	*C*_0,3_	*C*_1,3_	*C*_2,3_	*C*_3,3_	*C*_4,3_	...	C_*l*,3_
*C*	*C*_0,4_	*C*_1,4_	*C*_2,4_	*C*_3,4_	*C*_4,4_	...	*C*_*l*,4_

A count of nucleotides *A,T,G,C *at each position *K *= 1..*l *in all the sequences of the data set. *K *= 0 denotes the background count.

Q0,j=C0,j∑J∈{A,T,G,C}C0,J     (1)
 MathType@MTEF@5@5@+=feaafiart1ev1aaatCvAUfKttLearuWrP9MDH5MBPbIqV92AaeXatLxBI9gBaebbnrfifHhDYfgasaacH8akY=wiFfYdH8Gipec8Eeeu0xXdbba9frFj0=OqFfea0dXdd9vqai=hGuQ8kuc9pgc9s8qqaq=dirpe0xb9q8qiLsFr0=vr0=vr0dc8meaabaqaciaacaGaaeqabaqabeGadaaakeaacqWGrbqudaWgaaWcbaGaeGimaaJaeiilaWIaemOAaOgabeaakiabg2da9maalaaabaGaem4qam0aaSbaaSqaaiabicdaWiabcYcaSiabdQgaQbqabaaakeaadaaeqaqaaiabdoeadnaaBaaaleaacqaIWaamcqGGSaalcqWGkbGsaeqaaaqaaiabdQeakjabgIGiolabcUha7jabdgeabjabcYcaSiabdsfaujabcYcaSiabdEeahjabcYcaSiabdoeadjabc2ha9bqab0GaeyyeIuoaaaGccaWLjaGaaCzcamaabmaabaGaeGymaedacaGLOaGaayzkaaaaaa@4D26@

Qk,j=Ck,j+bjt+∑J∈{A,T,G,C}bJ     (2)
 MathType@MTEF@5@5@+=feaafiart1ev1aaatCvAUfKttLearuWrP9MDH5MBPbIqV92AaeXatLxBI9gBaebbnrfifHhDYfgasaacH8akY=wiFfYdH8Gipec8Eeeu0xXdbba9frFj0=OqFfea0dXdd9vqai=hGuQ8kuc9pgc9s8qqaq=dirpe0xb9q8qiLsFr0=vr0=vr0dc8meaabaqaciaacaGaaeqabaqabeGadaaakeaacqWGrbqudaWgaaWcbaGaem4AaSMaeiilaWIaemOAaOgabeaakiabg2da9maalaaabaGaem4qam0aaSbaaSqaaiabdUgaRjabcYcaSiabdQgaQbqabaGccqGHRaWkcqWGIbGydaWgaaWcbaGaemOAaOgabeaaaOqaaiabdsha0jabgUcaRmaaqababaGaemOyai2aaSbaaSqaaiabdQeakbqabaaabaGaemOsaOKaeyicI4Saei4EaSNaemyqaeKaeiilaWIaemivaqLaeiilaWIaem4raCKaeiilaWIaem4qamKaeiyFa0habeqdcqGHris5aaaakiaaxMaacaWLjaWaaeWaaeaacqaIYaGmaiaawIcacaGLPaaaaaa@528F@

Eq. (1) shows the background frequency of each nucleotide. *bj *(and *b*_*J*_) is known as the Laplacian or Bayesian correction and is equal to d * *Q*_0, *j *_where *d *is some constant usually set to unity. Eq. (2) gives the weight assigned to the type of nucleotide at the *k*^*th *^position of the motif.

A Position Specific Scoring Matrix (PSSM) can be constructed from one set of instances in a given set of *t *sequences. From (1) and (2), it is obvious that the following relationship holds:

∑j∈{A,T,G,C}Qk,j=1∀k=0,1,2,...l     (3)
 MathType@MTEF@5@5@+=feaafiart1ev1aaatCvAUfKttLearuWrP9MDH5MBPbIqV92AaeXatLxBI9gBaebbnrfifHhDYfgasaacH8akY=wiFfYdH8Gipec8Eeeu0xXdbba9frFj0=OqFfea0dXdd9vqai=hGuQ8kuc9pgc9s8qqaq=dirpe0xb9q8qiLsFr0=vr0=vr0dc8meaabaqaciaacaGaaeqabaqabeGadaaakeaafaqabeqacaaabaWaaabuaeaacqWGrbqudaWgaaWcbaGaem4AaSMaeiilaWIaemOAaOgabeaaaeaacqWGQbGAcqGHiiIZcqGG7bWEcqWGbbqqcqGGSaalcqWGubavcqGGSaalcqWGhbWrcqGGSaalcqWGdbWqcqGG9bqFaeqaniabggHiLdGccqGH9aqpcqaIXaqmaeaacqGHaiIicqWGRbWAcqGH9aqpcqaIWaamcqGGSaalcqaIXaqmcqGGSaalcqaIYaGmcqGGSaalcqGGUaGlcqGGUaGlcqGGUaGlcqWGSbaBaaGaaCzcaiaaxMaadaqadaqaaiabiodaZaGaayjkaiaawMcaaaaa@531A@

For a given *k *value in (3), each *Q *can be represented in terms of the other three variables. Since the length of the motif is *l*, the final objective function (i.e. the information content score) would contain 3*l *independent variables. It should be noted that even if there are 4*l *variables in total, the parameter space will contain only 3*l *independent variables because of the constraints obtained from (3). Thus, the constraints help in reducing the dimensionality of the search problem.

To obtain the information content (IC) score, every possible *l - mer *in each of the *t *sequences must be examined. This is done so by multiplying the respective *Q*_*i*, *j*_/*Q*_0, *j *_dictated by the nucleotides and their respective positions within the *l - mer*. Only the highest scoring *l - mer *in each sequence is noted and kept as part of the alignment. The total score is the sum of all the best (logarithmic) scores in each sequence.

A(Q)=∑i=1tlog(A)i=∑i=1tlog(∏k=1lQk,jQb)i     (4)
 MathType@MTEF@5@5@+=feaafiart1ev1aaatCvAUfKttLearuWrP9MDH5MBPbIqV92AaeXatLxBI9gBaebbnrfifHhDYfgasaacH8akY=wiFfYdH8Gipec8Eeeu0xXdbba9frFj0=OqFfea0dXdd9vqai=hGuQ8kuc9pgc9s8qqaq=dirpe0xb9q8qiLsFr0=vr0=vr0dc8meaabaqaciaacaGaaeqabaqabeGadaaakeaacqWGbbqqcqGGOaakcqWGrbqucqGGPaqkcqGH9aqpdaaeWbqaaGqaciab=XgaSjab=9gaVjab=DgaNjabcIcaOiabdgeabjabcMcaPmaaBaaaleaacqWGPbqAaeqaaaqaaiabdMgaPjabg2da9iabigdaXaqaaiabdsha0bqdcqGHris5aOGaeyypa0ZaaabCaeaacqWFSbaBcqWFVbWBcqWFNbWzdaqadaqaamaarahabaWaaSaaaeaacqWGrbqudaWgaaWcbaGaem4AaSMaeiilaWIaemOAaOgabeaaaOqaaiabdgfarnaaBaaaleaacqWGIbGyaeqaaaaaaeaacqWGRbWAcqGH9aqpcqaIXaqmaeaacqWGSbaBa0Gaey4dIunaaOGaayjkaiaawMcaaaWcbaGaemyAaKMaeyypa0JaeGymaedabaGaemiDaqhaniabggHiLdGcdaWgaaWcbaGaemyAaKgabeaakiaaxMaacaWLjaWaaeWaaeaacqaI0aanaiaawIcacaGLPaaaaaa@629A@

where *Q*_*k*, *j*_/*Q*_*b *_represents the ratio of the nucleotide probability to the corresponding background probability. *Log*(*A*)_*i *_is the score at each individual *i*^*th *^sequence. In equation (4), we see that *A *is composed of the product of the weights for each individual position *k*. We consider this to be the Information Content (IC) score which we would like to maximize. *A*(*Q*) is the non-convex 3*l *dimensional continuous function for which the global maximum corresponds to the best possible motif in the dataset. EM refinement performed at the end of a combinatorial approach has the disadvantage of converging to a local optimal solution [22]. Our method improves the procedure for refining motif by understanding the details of the stability boundaries and by trying to escape out of the convergence region of the EM algorithm.

### 3.2 Hessian Computation and Dynamical System for the Scoring Function

In order to present our algorithm, we have defined the dynamical system corresponding to the log-likelihood function and the PSSM. The key contribution of the paper is the development of this nonlinear dynamical system which will enable us to realize the geometric and dynamic nature of the likelihood surface by allowing us to understand the topology and convergence behaviour of any given subspace on the surface. We construct the following *gradient system *in order to locate critical points of the objective function (4):

Q˙
 MathType@MTEF@5@5@+=feaafiart1ev1aaatCvAUfKttLearuWrP9MDH5MBPbIqV92AaeXatLxBI9gBaebbnrfifHhDYfgasaacH8akY=wiFfYdH8Gipec8Eeeu0xXdbba9frFj0=OqFfea0dXdd9vqai=hGuQ8kuc9pgc9s8qqaq=dirpe0xb9q8qiLsFr0=vr0=vr0dc8meaabaqaciaacaGaaeqabaqabeGadaaakeaacuWGrbqugaGaaaaa@2DE0@(*t*) = -∇ *A*(*Q*)     (5)

One can realize that this transformation preserves all of the critical points [20]. Now, we will describe the construction of the gradient system and the Hessian in detail. In order to reduce the dominance of one variable over the other, the values of each of the nucleotides that belong to the consensus pattern at the position *k *will be represented in terms of the other three nucleotides in that particular column. Let *P*_*ik*_denote the *k*^*th *^position in the segment *P*_*i*_. This will also minimize the dominance of the eigenvector directions when the Hessian is obtained. The variables in the scoring function are transformed into new variables described in Table 2. Thus, Eq. (4) can be rewritten in terms of the 3*l *variables as follows:

**Table 2:** Position Weight Matrix. A count of nucleotides *j *∈ {*A*, *T*, *G*, *C*} at each position *k *= 1..*l *in all the sequences of the data set. *C*_*k *_is the *k*^*th *^nucleotide of the consensus pattern which represents the nucleotide with the highest value in that column. Let the consensus pattern be GACT...G and *b*_*j *_be the background.

*j*	*k = b*	*k *= 1	*k *= 2	*K *= 3	*k *= 4	...	*k = l*
*A*	*b*_*A*_	*w*_1_	*C*_2_	*w*_7_	*w*_10_	...	*w*_3*l*-2_
*T*	*b*_*T*_	*w*_2_	*w*_4_	*w*_8_	*C*_4_	...	*w*_3*l*-1_
*G*	*b*_*G*_	*C*_1_	*w*_5_	*w*_9_	*w*_11_	...	*C*_*l*_
*C*	*b*_*C*_	*W*_3_	*w*_6_	*C*_3_	*w*_12_	...	*W*_3*l*_

A(Q)=∑i=1t∑k=1llog fik(w3k−2,w3k−1,w3k)i     (6)
 MathType@MTEF@5@5@+=feaafiart1ev1aaatCvAUfKttLearuWrP9MDH5MBPbIqV92AaeXatLxBI9gBaebbnrfifHhDYfgasaacH8akY=wiFfYdH8Gipec8Eeeu0xXdbba9frFj0=OqFfea0dXdd9vqai=hGuQ8kuc9pgc9s8qqaq=dirpe0xb9q8qiLsFr0=vr0=vr0dc8meaabaqaciaacaGaaeqabaqabeGadaaakeaacqWGbbqqcqGGOaakcqWGrbqucqGGPaqkcqGH9aqpdaaeWbqaamaaqahabaacbiGae8hBaWMae83Ba8Mae83zaCgaleaacqWGRbWAcqGH9aqpcqaIXaqmaeaacqWGSbaBa0GaeyyeIuoaaSqaaiabdMgaPjabg2da9iabigdaXaqaaiabdsha0bqdcqGHris5aOGaeeiiaaIaemOzay2aaSbaaSqaaiabdMgaPjabdUgaRbqabaGccqGGOaakcqWG3bWDdaWgaaWcbaGaeG4mamJaem4AaSMaeyOeI0IaeGOmaidabeaakiabcYcaSiabdEha3naaBaaaleaacqaIZaWmcqWGRbWAcqGHsislcqaIXaqmaeqaaOGaeiilaWIaem4DaC3aaSbaaSqaaiabiodaZiabdUgaRbqabaGccqGGPaqkdaWgaaWcbaGaemyAaKgabeaakiaaxMaacaWLjaWaaeWaaeaacqaI2aGnaiaawIcacaGLPaaaaaa@6150@

where *f*_*ik *_can take the values {*w*_3*k*-2_*, w*_3*k*-1_, *w*_3*k*_, 1 - (*w*_3*k*-2 _+ *w*_3*k*-1 _+ *w*_3*k*_)} depending on the *P*_*ik *_value. The first derivative of the scoring function is a one dimensional vector with 3*l *elements.

∇A=[∂A∂w1∂A∂w2∂A∂w3....∂A∂w3l]T     (7)
 MathType@MTEF@5@5@+=feaafiart1ev1aaatCvAUfKttLearuWrP9MDH5MBPbIqV92AaeXatLxBI9gBaebbnrfifHhDYfgasaacH8akY=wiFfYdH8Gipec8Eeeu0xXdbba9frFj0=OqFfea0dXdd9vqai=hGuQ8kuc9pgc9s8qqaq=dirpe0xb9q8qiLsFr0=vr0=vr0dc8meaabaqaciaacaGaaeqabaqabeGadaaakeaacqGHhis0cqWGbbqqcqGH9aqpdaWadaqaamaalaaabaGaeyOaIyRaemyqaeeabaGaeyOaIyRaem4DaC3aaSbaaSqaaiabigdaXaqabaaaaOWaaSaaaeaacqGHciITcqWGbbqqaeaacqGHciITcqWG3bWDdaWgaaWcbaGaeGOmaidabeaaaaGcdaWcaaqaaiabgkGi2kabdgeabbqaaiabgkGi2kabdEha3naaBaaaleaacqaIZaWmaeqaaaaakiabc6caUiabc6caUiabc6caUiabc6caUmaalaaabaGaeyOaIyRaemyqaeeabaGaeyOaIyRaem4DaC3aaSbaaSqaaiabiodaZiabdYgaSbqabaaaaaGccaGLBbGaayzxaaWaaWbaaSqabeaacqWGubavaaGccaWLjaGaaCzcamaabmaabaGaeG4naCdacaGLOaGaayzkaaaaaa@5671@

and each partial derivative is given by

∂A∂wp=∑i=1t∂fip∂wpfik(w3k−2,w3k−1,w3k)     (8)
 MathType@MTEF@5@5@+=feaafiart1ev1aaatCvAUfKttLearuWrP9MDH5MBPbIqV92AaeXatLxBI9gBaebbnrfifHhDYfgasaacH8akY=wiFfYdH8Gipec8Eeeu0xXdbba9frFj0=OqFfea0dXdd9vqai=hGuQ8kuc9pgc9s8qqaq=dirpe0xb9q8qiLsFr0=vr0=vr0dc8meaabaqaciaacaGaaeqabaqabeGadaaakeaadaWcaaqaaiabgkGi2kabdgeabbqaaiabgkGi2kabdEha3naaBaaaleaacqWGWbaCaeqaaaaakiabg2da9maaqahabaWaaSaaaeaadaWcaaqaaiabgkGi2kabdAgaMnaaBaaaleaacqWGPbqAcqWGWbaCaeqaaaGcbaGaeyOaIyRaem4DaC3aaSbaaSqaaiabdchaWbqabaaaaaGcbaGaemOzay2aaSbaaSqaaiabdMgaPjabdUgaRbqabaGccqGGOaakcqWG3bWDdaWgaaWcbaGaeG4mamJaem4AaSMaeyOeI0IaeGOmaidabeaakiabcYcaSiabdEha3naaBaaaleaacqaIZaWmcqWGRbWAcqGHsislcqaIXaqmaeqaaOGaeiilaWIaem4DaC3aaSbaaSqaaiabiodaZiabdUgaRbqabaGccqGGPaqkaaaaleaacqWGPbqAcqGH9aqpcqaIXaqmaeaacqWG0baDa0GaeyyeIuoakiaaxMaacaWLjaWaaeWaaeaacqaI4aaoaiaawIcacaGLPaaaaaa@614C@

∀*p *= 1, 2 ... 3*l and k = round*(*p*/3)*+ *1

The Hessian ∇^2^*A *is a block diagonal matrix of block size 3 × 3. For a given sequence, the entries of the 3 × 3 block will be the same if that nucleotide belongs to the consensus pattern (*C*_*k*_). The gradient system is mainly obtained for enabling us to identify the stability boundaries and stability regions on the likelihood surface. The theoretical details of these concepts are published in [20]. The stability region of each local maximum is an approximate convergence zone of the EM algorithm. If we can identify all the saddle points on the stability boundary of a given local maximum, then we will be able to find all the corresponding Tier-1 local maxima. Tier-1 local maximum is defined as the new local maximum that is connected to the original local maximum through one decomposition point. Similarly, we can define Tier-2 and Tier-k local maxima that will take 2 and k decomposition points respectively. However, finding every saddle point is computationally intractable and hence we have adopted a heuristic by generating the eigenvector directions of the PSSM at the local maximum. Also, for such a complicated likelihood function, it is not efficient to compute all saddle points on the stability boundary. Hence, one can obtain new local maxima by obtaining the *exit points *instead of the saddle points. The point along a particular direction where the function has the lowest value starting from the given local maximum is called the *exit point*. The next section details our approach and explains the different phases of our algorithm.

## 4 Novel Framework

Our framework consists of the following three phases:

• *Global phase *in which the promising solutions in the entire search space are obtained.

• *Refinement phase *where a local method is applied to the solutions obtained in the previous phase in order to refine the profiles.

• *Exit phase *where the exit points are computed and the Tier-1 and Tier-2 solutions are explored systematically.

In the global phase, a branch and bound search is performed on the entire dataset. All of the profiles that do not meet a certain threshold (in terms of a given scoring function) are eliminated in this phase. The promising patterns obtained are transformed into profiles and local improvements are made to these profiles in the refinement phase. The consensus pattern is obtained from each nucleotide that corresponds to the largest value in each column of the PSSM. The 3*l *variables chosen are the nucleotides that correspond to those that are not present in the consensus pattern. Because of the probability constraints discussed in the previous section, the largest weight can be represented in terms of the other three variables.

To solve (4), current algorithms begin at random initial alignment positions and attempt to converge to an alignment of *l - mers *in all of the sequences that maximize the objective function. In other words, the *l - mer *whose *log*(*A*)_*i *_is the highest (with a given PSSM) is noted in every sequence as part of the current alignment. During the maximization of *A*(*Q*) function, the probability weight matrix and hence the corresponding alignments of *l - mers *are updated. This occurs iteratively until the PSSM converges to the local optimal solution. The consensus pattern is obtained from the nucleotide with the largest weight in each position (column) of the PSSM. This converged PSSM and the set of alignments correspond to a local optimal solution. The exit phase where the neighborhood of the original solution is explored in a systematic manner is shown below:

**Input: **Local Maximum (A).

**Output: **Best Local Maximum in the neighborhood region.

Algorithm:

*Step 1: *Construct the PSSM for the alignments corresponding to the local maximum (A) using Eqs.(1) and (2).

*Step 2: *Calculate the eigenvectors of the Hessian matrix for this PSSM.

*Step 3: *Find exit points (*e*_1*i*_) on the practical stability boundary along each eigenvector direction.

*Step 4: *For each of the exit points, the corresponding Tier-1 local maxima (*a*_1*i*_) are obtained by applying the EM algorithm after the ascent step.

*Step 5: *Repeat this process for promising Tier-1 solutions to obtain Tier-2 (*a*_2*j*_) local maxima.

*Step 6: *Return the solution that gives the maximum information content score of {*A*, *a*_1*i*_, *a*_2*j*_}.

**Figure 2:**
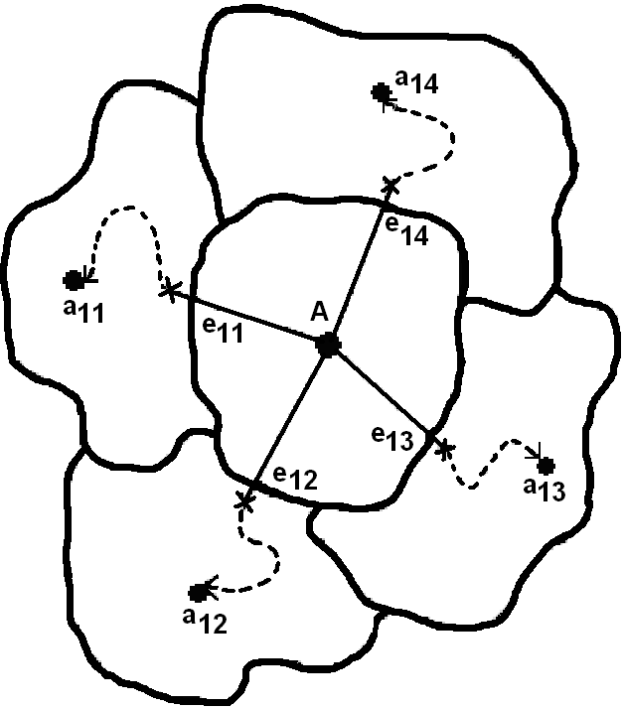
Diagram illustrates the exit point method of escaping from the original solution (*A*) to the neighborhood local optimal solutions (*a*_1*i*_) through the corresponding exit points (*e*_1*i*_). The dotted lines indicate the local convergence of the EM algorithm.

Fig. 2 illustrates the exit point method. To escape out of this local optimal solution, our approach requires the computation of a Hessian matrix (i.e. the matrix of second derivatives) of dimension (3*l*)^2 ^and the 3*l *eigenvectors of the Hessian. The main reasons for choosing the eigenvectors of the Hessian as search directions are:

• Computing the eigenvectors of the Hessian is related to finding the directions with extreme values of the second derivatives, i.e., directions of extreme normal-to-isosurface change.

• The eigenvectors of the Hessian will form the basis vectors for the search directions. Any other search direction can be obtained by a linear combination of these directions.

• This will make our algorithm deterministic since the eigenvector directions are always unique.

**Figure 3:**
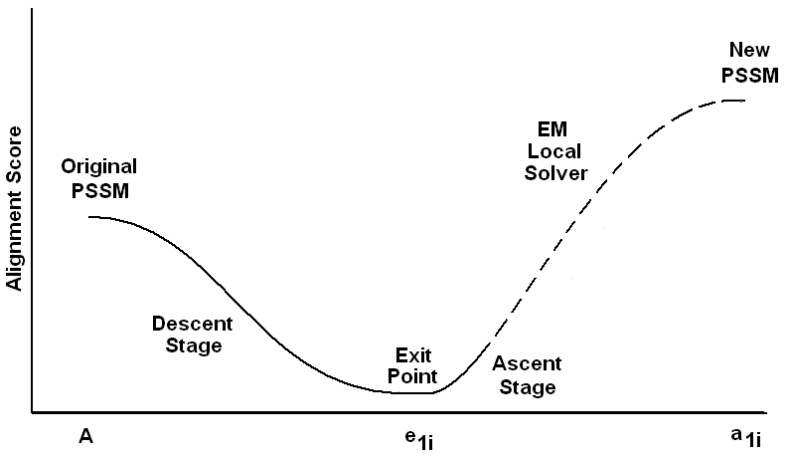
A summary of escaping out of the local optimum to the neighborhood local optimum. Observe the corresponding trend of *A*(*Q*) at each step.

The value of the objective function is evaluated along these eigenvector directions with some small step size increments. Since the starting position is a local optimal solution, one will see a steady decline in the function value during the initial steps; we call this the *descent stage*. Since the Hessian is obtained only once during the entire procedure, it is more efficient compared to Newton's method where an approximate Hessian is obtained for every iteration. After a certain number of evaluations, there may be an increase in the value indicating that the current point is out of the stability region of the local maximum. Once the exit point has been reached, few more evaluations are made in the direction of the same eigenvector to ensure that one has left the original stability region. This procedure is clearly shown in Fig 3. Applying the local method directly from the exit point may give the original local maximum. The ascent stage is used to ensure that the new guess is in a different convergence zone. Hence, given the best local maximum obtained using any current local methods, this framework allows us to systematically escape out of the local maximum to explore surrounding local maxima. The complete algorithm is shown below :

**Input: **The DNA sequences, length of the motif (1), Maximum Number of Mutations (d)

**Output: **Motif (s)

Algorithm:

*Step 1: *Given the sequences, apply Random Projection algorithm to obtain different set of alignments.

*Step 2: *Choose the promising buckets and apply EM algorithm to refine these alignments.

*Step 3: *Apply the exit point method to obtain nearby promising local optimal solutions.

*Step 4: *Report the consensus pattern that corresponds to the best alignments and their corresponding PSSM.

The new framework can be treated as a hybrid approach between global and local methods. It differs from traditional local methods by computing multiple local solutions in the neighborhood region in a systematic manner. It differs from global methods by working completely in the profile space and searching a subspace efficiently in a deterministic manner. For a given non-convex function, there is a massive number of convergence regions that are very close to each other and are separated from one another in the form of different basins of attraction. These basins are effectively modeled by the concept of stability regions.

## 5 Implementation Details

Our program was implemented on Red Hat Linux version 9 and runs on a Pentium IV 2.8 GHz machine. The core algorithm that we have implemented is *XP_EM *described in Algorithm 1. *XP_EM *obtains the initial alignments and the original data sequences along with the length of the motif. It returns the best motif that is obtained in the neighboring region of the sequences. This procedure constructs the PSSM, performs EM refinement, and then computes the Tier-1 and Tier-2 solutions by calling the procedure *Next_Tier*. The eigenvectors of the Hessian were computed using the source code obtained from [23]. *Next_Tier *takes a PSSM as an input and computes an array of PSSMs corresponding to the next tier local maxima using the exit point methodology.

**Algorithm 1 **Motif *XP_EM*(*init_aligns*, *seqs*, *l*)

   *PSSM *= *Construct_PSSM*(*init_aligns*)

   *New_PSSM *= *Apply_EM*(*PSSM*, *seqs*)

   *TIER*1 = *Next-Tier*(*seqs*, *New_PSSM*, *l*)

   **for ***i *= 1 to 3*l ***do**

      **if ***TIER*1[*i*] < > *zeros*(4*l*) **then**

         *TIER*2[*i*][ ] = *Next_Tier*(*seqs*, *TIER*1[*i*], *l*)

      **end if**

   **end for**

   Return *best*(*PSSM*, *TIER*1, *TIER*2)

Given a set of initial alignments, Algorithm 1 will find the best possible motif in the neighborhood space of the profiles. Initially, a PSSM is computed using *construct_PSSM *from the given alignments. The procedure *Apply_EM *will return a new PSSM that corresponds to the alignments obtained after the EM algorithm has been applied to the initial PSSM. The details of the procedure *Next_Tier *are given in Algorithm 2. From a given local solution (or PSSM), *Next_Tier *will compute all the 3*l *new *PSSM*s in the neighborhood of the given local optimal solution. The second tier patterns are obtained by calling the *Next_Tier *from the first tier solutions. Sometimes, New PSSMs might not be obtained for certain search directions. In those cases, a zero vector of length 4*l *is returned. Only those new PSSMs which do not have this value will be used for any further processing. Finally, the pattern with the highest score amongst all the PSSMs is returned.

The procedure *Next_Tier *takes a PSSM, applies the Exit-point method and computes an array of PSSMs that corresponds to the next tier local optimal solutions. The procedure *eval *evaluates the scoring function for the PSSM using (4). The procedures *Construct_Hessian *and *Compute_EigVec *compute the Hessian matrix and the eigenvectors respectively. *MAX_iter *indicates the maximum number of uphill evaluations that are required along each of the eigenvector directions. The neighborhood PSSMs will be stored in an array variable *PSSMs*[ ]. The original PSSM is updated with a small step until an exit point is reached or the number of iterations exceeds the *MAX_Iter *value. If the exit point is reached along a particular direction, some more iterations are run to guarantee that the PSSM has exited the original stability region and has entered a new one. The EM algorithm is then used during this ascent stage to obtain a new PSSM. For the sake of completeness, the entire algorithm has been shown in this section. However, during the implementation, several heuristics have been applied to reduce the running time of the algorithm. For example, if the first tier solution is not very promising, it will not be considered for obtaining the corresponding second tier solutions.

**Algorithm 2 **PSSMs[ ] *Next_Tier*(*seqs*, *PSSM*, *l*)

   *Score = eval*(*PSSM*)

   *Hess = Construct_Hessian*(*PSSM*)

   *Eig*[ ] = *Compute_EigVec*(*Hess*)

   *MAX_Iter *= 100

   **for ***k *= 1 to 3*l ***do**

      *PSSMs*[*k*] = *PSSM Count *= 0

      *Old_Score = Score ep_reached = FALSE*

      **while **(! *ep_reached*) && (*Count *<*MAX_Iter*) **do**

         *PSSMs*[*k*] = *update*(*PSSMs*[*k*], *Eig*[*k*], *step*)

         *Count = Count *+ 1

         *New_Score = eval*(*PSSMs*[*k*])

         **if **(*New-Score *> *Old-Score*) **then**

            *ep_reached = TRUE*

         **end if**

         *Old_Score = New_Score*

      **end while**

      **if ***count < MAX_Iter ***then**

         *PSSMs*[*k*] = *update*(*PSSMs*[*k*], *Eig*[*k*], *ASC*)

         *PSSMs*[*k*] = *Apply_EM*(*PSSMs*[*k*], *Seqs*)

      **else**

         *PSSMs*[*k*] = *zeros*(4*l*)

      **end if**

   **end for**

   **Return ***PSSMs*[ ]

**Figure 4:**
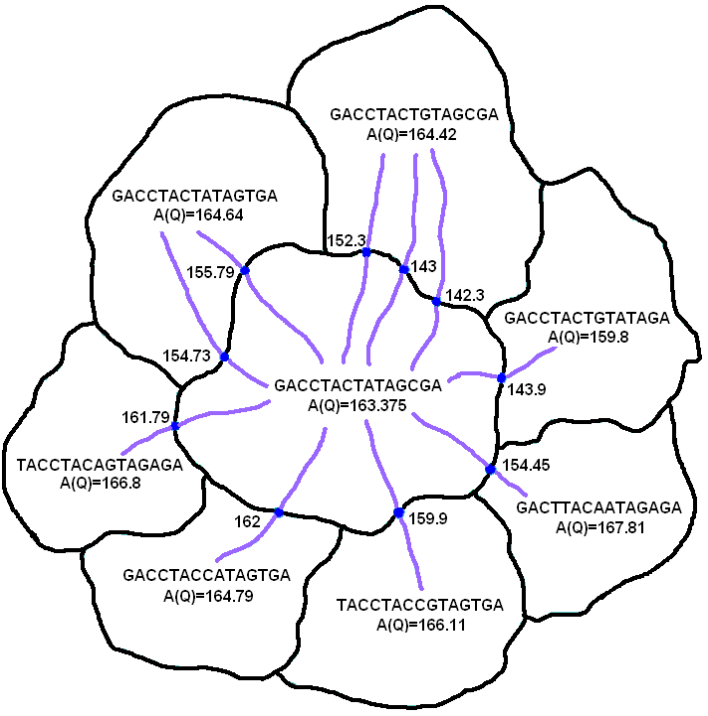
2-D illustration of first tier improvements in a 3*l *dimensional objective function. The original local maximum has a score of 163.375. The various Tier-1 solutions are plotted and the one with highest score (167.81) is chosen.

The initial alignments are converted into the profile space and a PSSM is constructed. The PSSM is updated (using the EM algorithm) until the alignments converge to a local optimal solution. The Exit-point methodology is then employed to escape out of this local optimal solution to compute nearby first tier local optimal solutions. This process is then repeated on promising first tier solutions to obtain second tier solutions. As shown in Fig. 4, from the original local optimal solution, various exit points and their corresponding new local optimal solutions are computed along each eigenvector direction. Sometimes two directions may yield the same local optimal solution. This can be avoided by computing the saddle point corresponding to the exit point on the stability boundary [24]. There can be many exit points, but there will only be a unique saddle point corresponding to the new local minimum. However, in high dimensional problems, this is not very efficient. Hence, we have chosen to compute the exit points. For computational efficiency, the Exit-point approach is only applied to promising initial alignments (i.e. random starts with higher Information Content score). Therefore, a threshold *A*(*Q*) score is determined by the average of the three best first tier scores after 10–15 random starts; any current and future first tier solution with scores greater than the threshold is considered for further analysis. Additional random starts are carried out in order to aggregate at least ten first tier solutions. The Exit-point method is repeated on all first tier solutions above a certain threshold to obtain second tier solutions.

## 6 Experimental Results

Experiments were performed on both synthetic data and real data. Two different methods were used in the global phase: random start and random projection. The main purpose of this paper is not to demonstrate that our algorithm can outperform the existing motif finding algorithms. Rather, the main work here focuses on improving the results that are obtained from other efficient algorithms. We have chosen to demonstrate the performance of our algorithm on the results obtained from the random projection method which is a powerful global method that has outperformed other traditional motif finding approaches like MEME, Gibbs sampling, WINNOWER, SP-STAR, etc. [1]. Since the comparison was already published, we mainly focus on the performance improvements of our algorithm as compared to the random projection algorithm. For the random start experiment, a total of *N *random numbers between 1 and (*t - l + *1) corresponding to initial set of alignments are generated. We then proceeded to evaluate our Exit-point methodology from these alignments.

### 6.1 Synthetic Datasets

The synthetic datasets were generated by implanting some motif instances into *t *= 20 sequences each of length *t*_*i *_= 600. Let *m *correspond to one full random projection + EM cycle. We have set *m *= 1 to demonstrate the efficiency of our approach. We compared the performance coefficient (PC) which gives a measure of the average performance of our implementation compared to that of Random Projection. The PC is given by :

PC=|K∩P||K∪P|     (9)
 MathType@MTEF@5@5@+=feaafiart1ev1aaatCvAUfKttLearuWrP9MDH5MBPbIqV92AaeXatLxBI9gBaebbnrfifHhDYfgasaacH8akY=wiFfYdH8Gipec8Eeeu0xXdbba9frFj0=OqFfea0dXdd9vqai=hGuQ8kuc9pgc9s8qqaq=dirpe0xb9q8qiLsFr0=vr0=vr0dc8meaabaqaciaacaGaaeqabaqabeGadaaakeaacqWGqbaucqWGdbWqcqGH9aqpdaWcaaqaamaaemaabaGaem4saSKaeyykICSaemiuaafacaGLhWUaayjcSdaabaWaaqWaaeaacqWGlbWscqGHQicYcqWGqbauaiaawEa7caGLiWoaaaGaaCzcaiaaxMaadaqadaqaaiabiMda5aGaayjkaiaawMcaaaaa@41D9@

where K is the set of the residue positions of the planted motif instances, and P is the corresponding set of positions predicted by the algorithm. Table 3 gives an overview of the performance of our method compared to the random projection algorithm on the (*l*, *d*) motif problem for different *l *and *d *values.

**Table 3:** Improvements in the Performance Coefficient.

Motif (l, d)	PC obtained using Random Projection	PC obtained using Exit-point method
(11,2)	20	20
(15,4)	14.875	17
(20,6)	12.667	18

The results of performance coefficient with *m *= 1 on synthetically generated sequences. The IC scores are not normalized and the perfect score is 20 since there are 20 sequences.

Our results show that by branching out and discovering multiple local optimal solutions, higher *m *values are not needed. A higher *m *value corresponds to more computational time because projecting the *l*-mers into *k*-sized buckets is a time consuming task. Using our approach, we can replace the need for randomly projecting *l*-mers repeatedly in an effort to converge to a global optimum by deterministically and systematically searching the solution space modeled by our dynamical system and improving the quality of the existing solutions. We can see that for higher length motifs, the improvements are more significant. Fig. 4 shows the Tier-1 solutions obtained from a given consensus pattern. Since the exit points are being used instead of saddle points, our method might sometimes find the same local optimal solution obtained before. As seen from the figure, the Tier-1 solutions can differ from the original pattern by more than just one nucleotide position. Also, the function value at the exit points is much higher than the original value.

**Figure 5:**
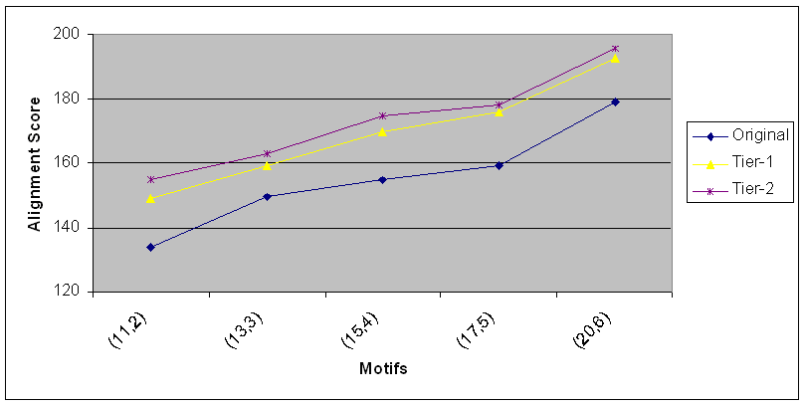
The average scores with the corresponding first tier and second tier improvements on synthetic data using the random starts with Exit-point approach with different (*l*, *d*) motifs.

As opposed to stochastic processes like mutations in genetic algorithms, our approach reduces the stochastic nature and obtains the nearby local optimal solutions systematically. Fig. 5 shows the performance of the Exit-point approach on synthetic data for different (*l*, *d*) motifs. The average scores of the ten best solutions obtained from random starts and their corresponding improvements in Tier-1 and Tier-2 are reported. One can see that the improvements become more prominent as the length of the motif is increased. Table 4 shows the best and worst of these top ten random starts along with the consensus pattern and the alignment scores.

**Table 4:** Improvements in the Information Content Scores.

(l, d)	Initial Pattern	Score	First Tier Pattern	Score	Second Tier Pattern	Score
(11,2)	AACGGTCGCAG	125.1	CCCGGTCGCTG	147.1	CCCGGGAGCTG	153.3
(11,2)	ATACCAGTTAC	145.7	ATACCAGTTTC	151.3	ATACCAGGGTC	153.6
(13,3)	CTACGGTCGTCTT	142.6	CCACGGTTGTCTC	157.8	CCTCGGGTTTGTC	158.7
(13,3)	GACGCTAGGGGGT	158.3	GAGGCTGGGCAGT	161.7	GACCTTGGGTATT	165.8
(15,4)	CCGAAAAGAGTCCGA	147.5	CCGCAATGACTGGGT	169.1	CCGAAAGGACTGCGT	176.2
(15,4)	TGGGTGATGCCTATG	164.6	TGGGTGATGCCTATG	166.7	TGAGAGATGCCTATG	170.4
(17,5)	TTGTAGCAAAGGCTAAA	143.3	CAGTAGCAAAGACTACC	173.3	CAGTAGCAAAGACTTCC	175.8
(17,5)	ATCGCGAAAGGTTGTGG	174.1	ATCGCGAAAGGATGTGG	176.7	ATTGCGAAAGAATGTGG	178.3
(20,6)	CTGGTGATTGAGATCATCAT	165.9	CAGATGGTTGAGATCACCTT	186.9	CATTTAGCTGAGTTCACCTT	194.9
(20,6)	GGTCACTTAGTGGCGCCATG	216.3	GGTCACTTAGTGGCGCCATG	218.8	CGTCACTTAGTCGCGCCATG	219.7

The consensus patterns and their corresponding scores of the original local optimal solution obtained from multiple random starts on the synthetic data. The best first tier and second tier optimal patterns and their corresponding scores are also reported.

**Figure 6:**
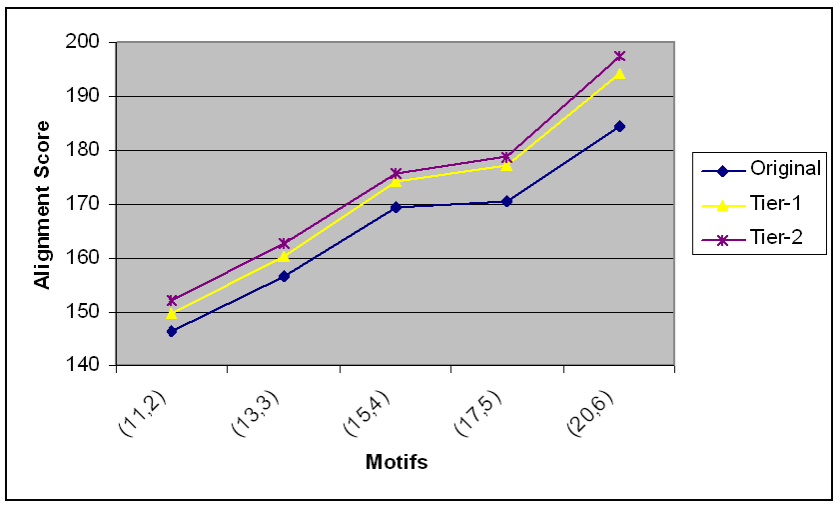
The average scores with the corresponding first tier and second tier improvements on synthetic data using the Random Projection with Exit-point approach with different (*l*, *d*) motifs.

With a few modifications, more experiments were conducted using the Random Projection method. The Random Projection method will eliminate non-promising regions in the search space and gives a number of promising sets of initial patterns. EM refinement is applied to only the promising initial patterns. Due to the robustness of the results, the Exit-point method is employed only on the top five local optima. The Exit-point method is again repeated on the top scoring first tier solutions to arrive at the second tier solutions. Fig. 6 shows the average alignment scores of the best random projection alignments and their corresponding improvements in tier-1 and tier-2 are reported. In general, the improvement in the first tier solutions are more significant than the improvements in the second tier solutions.

### 6.2 Real Datasets

Table 5 shows the results of the Exit-point methodology on real biological sequences. We have chosen *l *= 20 and *d *= 2. '*t*' indicates the number of sequences in the real data. For the biological samples taken from [1,12], the value *m *once again is the average number of random projection + EM cycles required to discover the motif. All other parameter values (like projection size *k *= 7 and threshold *s *= 4) are chosen to be the same as those used in the Random projection paper [1]. All of the motifs were recovered with *m *= 1 using the Exit-point strategy. The Random Projection algorithm alone needed *multiple cycles *(m = 8 in some cases and m = 15 in others) in order to retrieve the correct motif. This elucidates the fact that global methods can only be used to a certain extent and should be combined with refined local heuristics in order to obtain better efficiency. Since the random projection algorithm has outperformed other prominent motif finding algorithms like SP-STAR, WINNOWER, Gibbs sampling etc., we did not repeat the same experiments that were conducted in [1]. Running one cycle of random projection + EM is much more expensive computationally. The main advantage of our strategy comes from the deterministic nature of our algorithm in refining motifs.

**Table 5:** Results on real datasets. Results of Exit-point method on biological samples. The real motifs were obtained in all the six cases using the Exit-point framework.

**Sequence**	**Sample Size**	**t**	**Best (20,2) Motif**	**Reference Motif**
E. coli CRP	1890	18	TGTGAAATAGATCACATTTT	TGTGANNNNGNTCACA
preproinsulin	7689	4	GGAAATTGCAGCCTCAGCCC	CCTCAGCCC
DHFR	800	4	CTGCAATTTCGCGCCAAACT	ATTTCNNGCCA
metallothionein	6823	4	CCCTCTGCGCCCGGACCGGT	TGCRCYCGG
c-fos	3695	5	CCATATTAGGACATCTGCGT	CCATATTAGAGACTCT
yeast ECB	5000	5	GTATTTCCCGTTTAGGAAAA	TTTCCCNNTNAGGAAA

Let the cost of applying EM algorithm for a given bucket be *f *and let the average number of buckets for a given projection be *b*. Then the running time of the Exit-point method will be *O*(*cbf*) where *c *is a constant that is linear in *l*-the length of the motif. If there were *m *projections, then cost of the random projection algorithm using restarts will be *O*(*mbf*). The two main advantages of using Exit-point strategy compared to random projection algorithm are :

• It avoids multiple random projections which often provide similar optimal motifs.

• It provides multiple optimal solutions in a promising region of a given bucket as opposed to a single solution provided by random projection algorithm.

## 7 Concluding Discussion

The Exit-point framework proposed in this paper broadens the search region in order to obtain an improved solution which may potentially correspond to a better motif. In most of the profile based algorithms, EM is used to obtain the nearest local optimum from a given starting point. In our approach, we consider the boundaries of these convergence regions and find the surrounding local optimal solutions based on the theory of stability regions. We have shown on both real and synthetic data sets that beginning from the EM converged solution, the Exit-point approach is capable of searching in the neighborhood regions for another solution with an improved information content score. This will often translate into finding a pattern with less Hamming distance from the resulting alignments in each sequence. Our approach has demonstrated an improvement in the score on all datasets that it was tested on. One of the primary advantages of the Exit-point methodology is that it can be used with different global and local methods. The main contribution of our work is to demonstrate the capability of this hybrid EM algorithm in the context of the motif finding problem. Our algorithm can potentially use any global method and improve its results efficiently.

From our results, we see that motif refinement stage plays a vital role and can yield accurate results deterministically. We would like to continue our work by combining other global methods available in the literature with existing local solvers like EM or GibbsDNA that work in continuous space. By following the example of [4], we may improve the chances of finding more promising patterns by combining our algorithm with different global and local methods.
